# Pharmacotherapy consideration of thrombolytic medications in COVID-19-associated ARDS

**DOI:** 10.1186/s40560-022-00625-4

**Published:** 2022-07-30

**Authors:** Shahideh Amini, Aysa Rezabakhsh, Javad Hashemi, Fatemeh Saghafi, Hossein Azizi, Antoni Sureda, Solomon Habtemariam, Hamid Reza Khayat Kashani, Zahra Hesari, Adeleh Sahebnasagh

**Affiliations:** 1 Department of Clinical Pharmacy, Faculty of Pharmacy, Tehran University of Medical Sciences, Tehran, Iran; 2 Cardiovascular Research Center, Tabriz University of Medical Sciences, Tabriz, Iran; 3 Department of Pathobiology and Laboratory Sciences, School of Medicine, North Khorasan University of Medical Sciences, Bojnurd, Iran; 4 Department of Clinical Pharmacy, Faculty of Pharmacy and Pharmaceutical Sciences Research Center, Shahid Sadoughi University of Medical Sciences, Yazd, Iran; 5 School of Medicine, Tehran University of Medical Sciences, Tehran, Iran; 6 Research Group On Community Nutrition and Oxidative Stress, University of the Balearic Islands, Palma, Spain; 7 CIBEROBN (Physiopathology of Obesity and Nutrition CB12/03/30038), Instituto de Salud Carlos III, Madrid, Spain; 8 Pharmacognosy Research Laboratories and Herbal Analysis Services, University of Greenwich, Central Avenue, Chatham-Maritime, Kent, ME4 4TB UK; 9 Imam Hossein Hospital, Shahid Beheshti University of Medical Sciences, Tehran, Iran; 10 Laboratory Sciences Research Center, Golestan University of Medical Sciences, Gorgan, Iran; 11 Clinical Research Center, Department of Internal Medicine, School of Medicine, North Khorasan University of Medical Sciences, Bojnurd, Iran

**Keywords:** COVID-19, ARDS, Coagulopathy, Thrombolytic therapy, Pharmacotherapy, Pro-inflammatory

## Abstract

**Background:**

In late 2019, the severe acute respiratory syndrome coronavirus 2 (SARS-CoV-2) which is responsible for coronavirus disease (COVID-19), was identified as the new pathogen to lead pneumonia in Wuhan, China, which has spread all over the world and developed into a pandemic. Despite the over 1 year of pandemic, due to the lack of an effective treatment plan, the morbidity and mortality of COVID-19 remains high. Efforts are underway to find the optimal management for this viral disease.

**Main body:**

SARS-CoV-2 could simultaneously affect multiple organs with variable degrees of severity, from mild to critical disease. Overproduction of pro-inflammatory mediators, exacerbated cellular and humoral immune responses, and coagulopathy such as Pulmonary Intravascular Coagulopathy (PIC) contributes to cell injuries. Considering the pathophysiology of the disease and multiple microthrombi developments in COVID-19, thrombolytic medications seem to play a role in the management of the disease. Beyond the anticoagulation, the exact role of thrombolytic medications in the management of patients with COVID-19-associated acute respiratory distress syndrome (ARDS) is not explicit. This review focuses on current progress in underlying mechanisms of COVID-19-associated pulmonary intravascular coagulopathy, the historical use of thrombolytic drugs in the management of ARDS, and pharmacotherapy considerations of thrombolytic therapy, their possible benefits, and pitfalls in COVID-19-associated ARDS.

**Conclusions:**

Inhaled or intravenous administration of thrombolytics appears to be a salvage therapy for severe ARDS associated with COVID-19 by prompt attenuation of lung injury. Considering the pathogenesis of COVID-19-related ARDS and mechanism of action of thrombolytic agents, thrombolytics appear attractive options in stable patients without contraindications.

## Background

Belonging to a large family of viruses called coronaviridae, coronaviruses mainly cause a broad array of respiratory symptoms ranging from the common cold to severe pneumonia [1]. It has been suggested that COVID-19 comprised of three critical clinicobiological phases. In the initial step, it is estimated that 50–80% of the infected patients are asymptomatic or pre-symptomatic. In this phase, the patients have no symptoms but their screening tests become positive at the time of diagnosis. Patients gradually become symptomatic during the 5.2 days, as the incubation period. They usually experience the symptoms of involvement of the pulmonary or gastrointestinal systems. In the propagating step as the second phase of COVID-19, the infection progresses to the lower respiratory track and other organs, such as heart, kidney, hematopoietic and central nervous systems. If proper therapeutic modalities are not taken or there is a specific background condition, the disease can progress to the complicating phase three. In this phase, the infection spreads to all organs of the body and cause various complications with high mortality rate, such as Acute Respiratory Distress Syndrome (ARDS), sepsis, multiple organ failure, and shock [2]. Overproduction of pro-inflammatory mediators, exacerbated cellular and humoral immune responses and considerable tissue damage contribute to what is known as the cytokine storm or cytokine release syndrome [3]. This over-activation of the immune system which is intended to increase viral clearance leads to a series of intra-tissue events that eventually cause overwhelming inflammation, Acute Lung Injury (ALI)/ARDS, or even death [4]. Notably, viremia has not been reported in COVID-19 and the direct role of the viral infection in the progression of the disease is negligible. Rather, the role of the three-headed dragon of inflammation, coagulation and fibrinolysis is noteworthy like other types of sepsis.

In COVID-19, the cytokine release syndrome induces coagulopathy and thrombotic complications, such as disseminated intravascular coagulation (DIC) and pulmonary intravascular coagulopathy (PIC). PIC generally presents itself as elevated levels of D-dimer, C-reactive protein (CRP), and ferritin, which have been associated with the increased mortality rate [5]. In COVID-19-associated PIC, pulmonary involvement is reported in all the positive cases of COVID-19 [6]. As part of the host defense responses, viral infection initiates the complex systemic inflammation that may lead to coagulation, thrombosis and microangiopathy [7]. Several reports on COVID-19 pathology indicated the presence of micro and macro-circulatory dysfunction, cerebrovascular events, myocardial infarction, systemic hypercoagulability and thromboembolic complications due to endotheliitis and endothelial dysfunction [8, 9].

Considering the underlying pathophysiology of the disease and multiple microthrombi development in COVID-19, thrombolytic medications appear to play a role in the management of the disease. In other words, considering the three critical clinicobiological phases mentioned for COVID-19, anti-thrombolytics seem to have a potential role in the complicating phase 3 of the disease. Currently, the exact role of thrombolytic therapy in the management of patients with COVID-19 is not explicit and, these drugs may be used off-label, alone or in combination with anticoagulant medications. This review is addressing a summary of COVID-19-associated pulmonary intravascular coagulopathy, discussion on the historical use of thrombolytic drugs in management of ARDS; and the pharmacotherapy considerations of thrombolytic therapy, and their possible benefits and pitfalls in COVID-19-associated ARDS.

### The respiratory pathogenesis of COVID-19

Following transmission, the SARS-CoV-2 replicates in the mucosal epithelium of the lower and upper respiratory tracts and causes pneumonia with no or mild symptoms. This is followed by a decline in viral levels and the action of the adaptive immune system. The immune response is dependent on T cells, with the participation of CD4 helper T cells. They contribute to the production of specific neutralizing antibodies, and cytotoxic CD8 cells which kill the virus-infected cells. However, if the immune protective responses fail to inhibit the viral replication and eliminate the infected cells, the disease could progress into a lethal disease with a high level of inflammation and respiratory dysfunction [10, 11]. Eventually, immune dysfunction and pulmonary edema induce ARDS which is the main cause of mortality in infected patients [12]. Despite the detection of SARS-CoV-2 in the clinical specimens obtained from the upper respiratory tract, the overexpression of angiotensin-converting enzyme 2 (ACE2), and transmembrane protease serine 2 (TMPRSS2) genes determine the nature of SARS-CoV-2 as an infection of the lower respiratory tract [13]. SARS-CoV-2 entry through the ACE2 receptors, located on the type II pneumocytes, is directly dependent on TMPRSS2 and endosomal cysteine proteases cathepsin B and L (CatB/L) [14]. TMPRSS2 is considered a necessary factor for cleaving the envelope-located S protein subunits to make simultaneous viral and cell membrane fusion and viral internalization into the pulmonary epithelium [14]. The high serum levels of pro-inflammatory biomarkers, including TNF-α, Interleukin 2 (IL-2), IL-7, IL-10, granulocyte colony-stimulating factor (G-CSF), C–X–C motif chemokine ligand 10 (CXCL10), monocyte chemotactic protein-1 (MCP-1), and macrophage inflammatory proteins-1α (MIP-1α) were all detected in hospitalized COVID-19 patients. The level of IL-6 in non-survivor patients is significantly higher than in survivor individuals [15]. This triggers the vascular permeability and ACE2 overexpression. Nuclear factor kappa B (NF-κB), signal transducer activation, and activator of transcription 3 (STAT3) signaling pathways play key role in the alveolar epithelial and endothelial cells for regulation of the IL-6-dependent machinery [16].

### The characteristics of pulmonary intravascular coagulopathy of COVID-19

The higher incidence of coagulation abnormalities and venous thromboembolic (VTE) in patients with severe COVID-19 has been shown to be attributed to the coagulopathy rather than direct injury by SARS-CoV-2 infection. Although other pathogens-induced coagulopathy shares similar features, coagulopathy in COVID-19 differs from DIC induced by other viral and bacterial infections [18, 19].

It has been shown that the characteristics of COVID-19-associated coagulopathy vary greatly from disseminated intravascular coagulation [20], so the new term of pulmonary intravascular coagulopathy (PIC) was first introduced by McGonagle et al. [6]. Marongiu et al. also described localized pulmonary intravascular coagulation as an alternative process in COVID-19 instead of PIC to emphasize on the distinction behind the COVID-19-associated coagulopathy [21]. Since COVID-19 induced hypercoagulation is concentrated in the pulmonary system rather than systemic coagulation disorder, the term PIC has been used specifically for the disease in various sources [20]. Consequently, pulmonary involvement is seen in 100% of patients with PIC linked to COVID-19 [6]. SARS-CoV-2 finds access to the respiratory system through ACE2 receptor on pneumocytes. Due to the distribution of pneumocyte on 90–95% of the alveolar surface of the peripheral lung, the chest imaging findings of severe COVID-19 patients described diffuse alveolar damage [6, 22].

An increase in D-dimer levels, which is an indicator of fibrinolysis, is related to both the coagulopathy and the severity of COVID-19. Nevertheless, despite the surge elevation in D-dimer, overt systemic disseminated intravascular coagulation has not been generally reported in patients with COVID-19-associated coagulopathy. Unlike DIC, in COVID-19, the plasma abnormalities of prothrombin time (PT), activated partial thromboplastin time (aPTT), and platelet count are rare. In fact, COVID-19 is characterized by hypercoagulability and a high risk of thrombosis rather than bleeding events. If local host responses are not able to suppress immunothrombosis, the immunothrombosis disseminates into systemic circulation, and cause systemic microvascular thrombosis, which is better known as a DIC [20, 23].

In both DIC and PIC, a marked elevation in the levels of D-dimer, CRP, ferritin, and cytokines in the blood has been observed. Despite the many similarities between DIC and PIC, one difference is that the pulmonary involvement is seen in about 50% of DIC patients, while pulmonary involvement is observed in 100% of positive cases of COVID-19 PIC [6]. Intrapulmonary microhemorrhage has been reported in patients with COVID-19. Thrombosis occurs mainly in the lung rather than other tissues. The elevated prothrombin time (PT) or activated partial thromboplastin time (aPTT) is usually observed in DIC. On the other side, a normal level or slightly increased PT and aPTT is the main characteristic of PIC induced by SARS-CoV2 [6, 19, 24].

As the virus enters into the respiratory system, SARS-CoV-2 infects the ACE2 receptor-expressing type II pneumocytes and vascular endothelial cells and causes immediate cell lysis [25]. Due to cell lysis, damage-associated molecular patterns (DAMPs) are released which activates the immune cells such as monocytes and macrophages. The production of pro-inflammatory cytokines by infected cells as well as immune cells, including IL-1, IL-6, TNF-α, and monocyte chemoattractant protein-1 (MCP-1), further elevates the inflammation level at the site of infection [26, 27]. This subsequently leads to the lysis of the cells, additional activation of endothelial cells, and the consequent increase in the procoagulant activity, and accumulation of fibrin deposits in pulmonary microcapillary venous vessels. The activated monocytes express tissue factor (TF) surface protein which, in turn, directly activates coagulation and induces fibrin deposits in pulmonary microcapillary vessels [28, 29]. Although a marked increase in plasminogen in the early stages of the disease is observed, it fails to breakdown the fibrin deposits possibly due to the presence of D-dimer and fibrin degradation product (FDP) through fibrinolysis [30, 31]. These findings showed that COVID-19 PIC is presented as prothrombotic rather than hemorrhagic complications [32, 33].

**Fig. 1:**
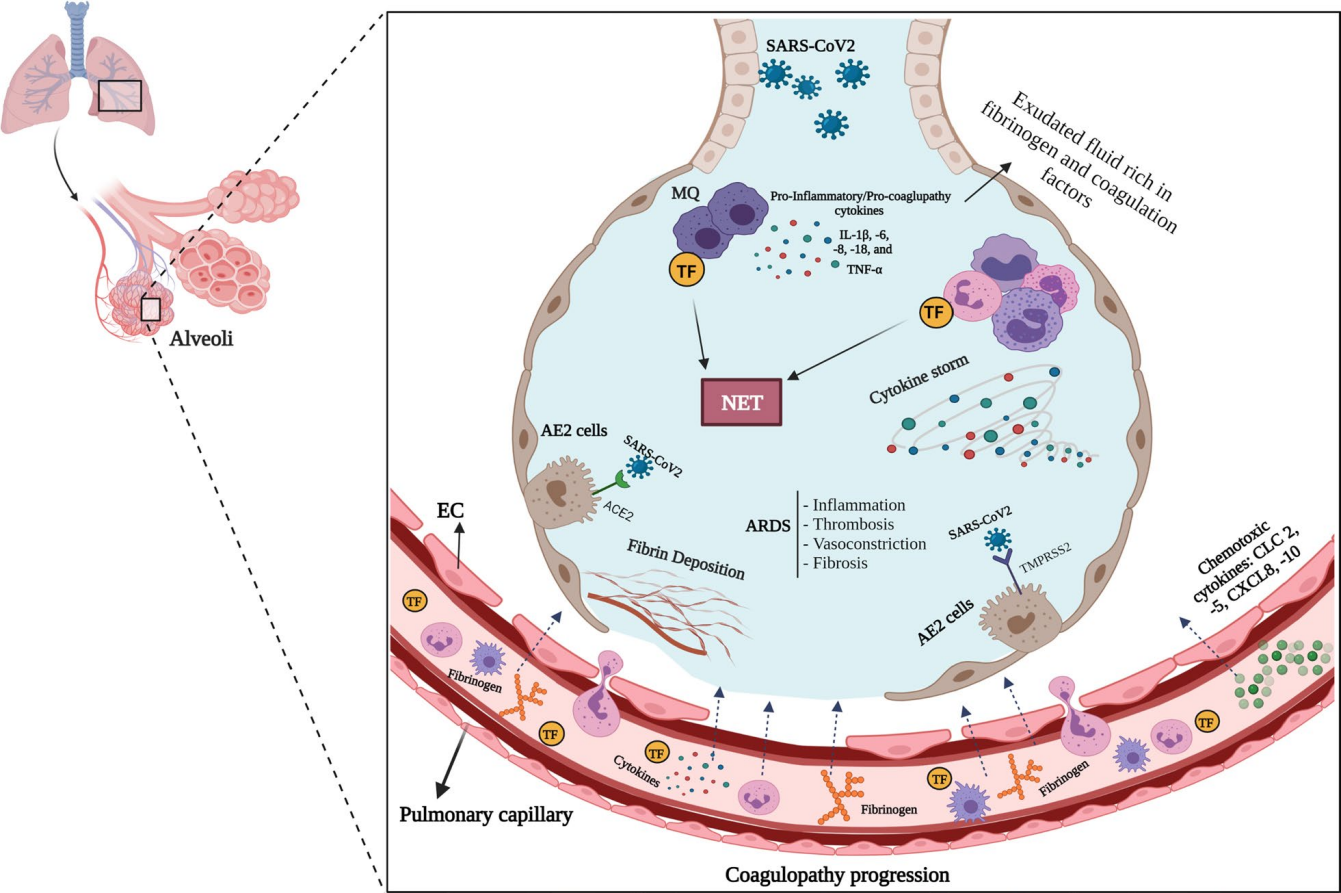
Micro- and macro-coagulopathy development during ARDS induced by COVID-19. *Created with BioRender.com

Along with the destructive role of imbalance in inflammatory cytokines, the components of endothelial cells, such as glycocalyx and antithrombin, are crucial for maintaining the normal hemostasis and vessel permeability as well as regulating the fibrinolysis [6]. During the severe infection, these components are of limited availability due to destruction by overexpressed heparanase produced by endothelial cells and matrix metalloproteinases (MMPs) produced by immune cells [34]. Endothelial cells dysfunction, which is further exacerbated by the cytokine storm, contributes to vasculopathy, hypoxia, and refractory ARDS [6]. Injured endothelial cells also play a critical role in microvascular thrombosis in COVID-19, since these cells produce ultra-large von Willebrand factor (VWF). VWF, which is physiologically cleaved by ADAMTS-13 (a disintegrin and metalloprotease with a thrombospondin type I motif-13) [35], increases the platelet-vessel wall interactions. However, a decrease in ADAMTS-13 level is observed in COVID-19 patients due to its consumption or proteolysis which results in platelet aggregation and deposition [36]. A massive release of tissue-type plasminogen activator (t-PA) has been also reported in injured endothelial cells leading to an increase in plasmin concentration [37]. Moreover, decreases in plasminogen activator inhibitor-1 (PAI-1), thrombomodulin, heparin sulfate, and antithrombin are observed in severely infected patients. PAI-1 is considered a biomarker for endothelial injury. It has been shown that its level increases in severe cases of COVID-19 [38]. An elevated level of plasmin and plasminogen facilitates the fibrinolysis and increases d-dimer and FDP in patients developing ARDS [6, 18, 27]. Furthermore, plasmin changes the extracellular matrix by affecting metalloproteinase, thereby facilitates the pulmonary capillary microleakage and edema [39]. The expression of tissue factor by immune cells (neutrophils, monocytes, and macrophages) further activates the coagulation cascade [40]. Figure 1 displays the micro and macro-coagulopathy development during the ARDS-induced by COVID-19.

Platelet adhesion, aggregation, and activation in response to the excess pro-inflammatory cytokines favor platelet thrombi formation in pulmonary microcapillary vessels leading to impairment in normal organ function [41].

Postmortem examinations have shown the presence of microvasculature platelet aggregation, thrombosis and microangiopathy in the small vessels and capillaries of the lungs which mainly attribute to mortality in patients with COVID-19. It is worth noting that the presence of thrombotic process in other organs such as the kidney, liver, pancreas or spleen is rare [42]. Although platelet and clot formation usually results in thrombocytopenia, intravascular hemolysis and thrombocytopenia are not the prominent clinical features of COVID-19 [27].

Ultimately, endothelial lesions mediated by cytokine storm can affect the integrity of the blood/air barrier, causing vascular permeability, alveolar edema, infiltration of leukocytes (neutrophils and macrophages) and hypoxia. Hypoxia further activates the coagulation cascade by inducing the thromboinflammatory positive feedback loop. These phenomenon finally develop pulmonary vascular thrombosis with a high incidence of deep vein thrombosis (DVT), pulmonary embolism (PE), and to a lesser extent, hemorrhagic complications in COVID-19 patients with ARDS symptoms [43].

### Historical use of medications affecting coagulation in the management of ARDS: animal and clinical studies

Since coagulopathy and thrombolysis are involved in the complex pathophysiology of ARDS, many attempts have been carried out to apply anticoagulant and antithrombotic therapies for patients with ARDS. The three coagulation pathways involved in the disease are (a) tissue factor pathway, (b) protein C pathway, and (c) plasminogen activator (PA) and inhibitor pathway. These pathways are promising targets for anticoagulation and antithrombotic therapies [44].

In a systematic review of preclinical and clinical studies, the effectiveness and safety of local anticoagulant agents, in nebulized formulation, including activated protein C, antithrombin, heparin and LMWH were evaluated in ALI. They concluded that the nebulized formulation of anticoagulation agents prevents coagulopathy and inflammation in the respiratory system in preclinical studies and ALI in human trials [45].

*Thrombolytic Medications (Plasminogen activators)* Thrombolytic medications, either urokinase-type (uPA) or tissue-type (t-PA), produced from endothelial and alveolar cells, are responsible for the conversion of plasminogen to plasmin. Three major classes of plasmin activators or thrombolytic drugs with almost similar pharmacokinetics properties are categorized into: (1) tissue plasminogen activator (t-PA), (2) SKA, and (3) UK compounds.

**Fig. 2:**
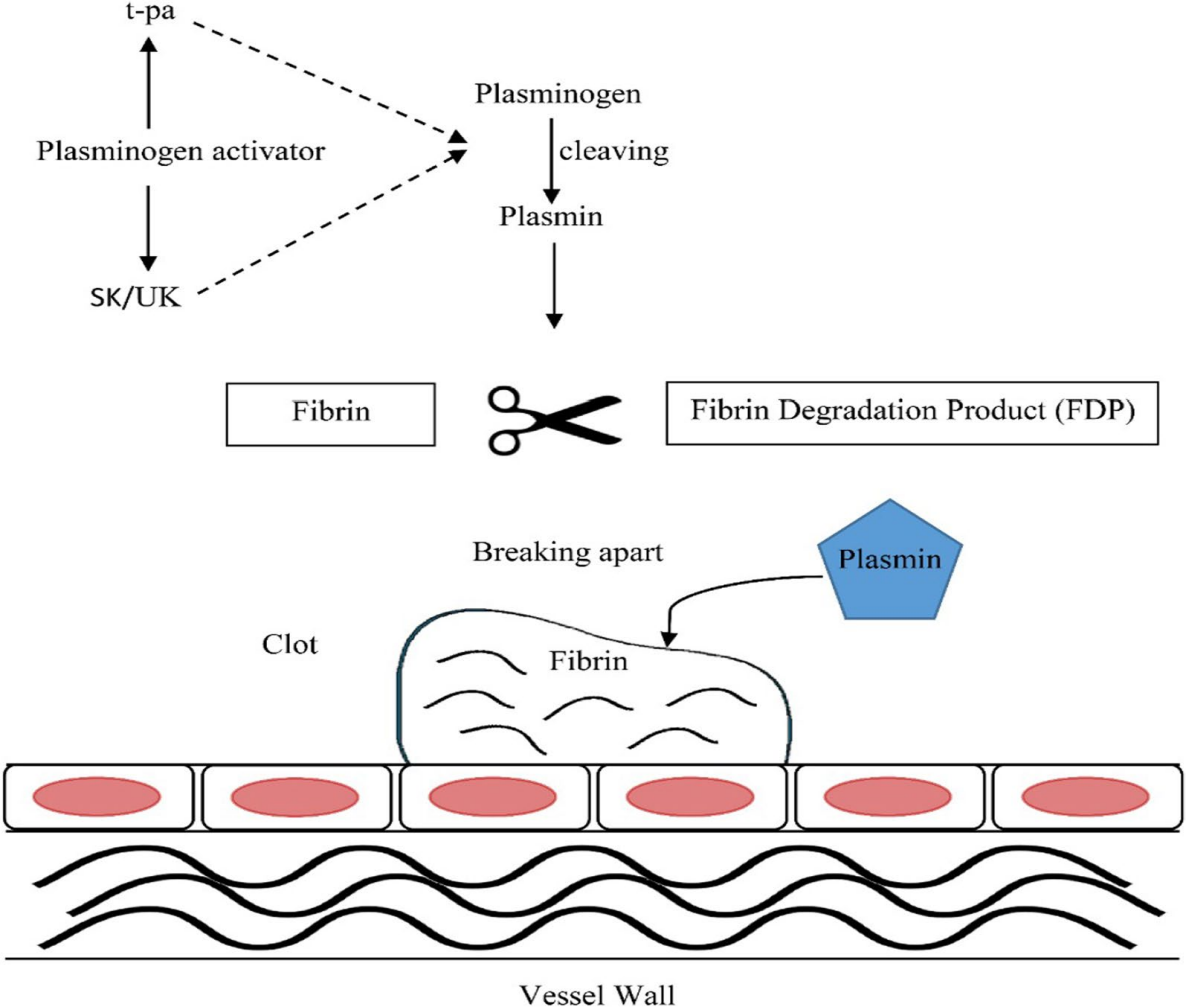
Mechanism of action of thrombolytic drugs

The mechanism of action of thrombolytic drugs is presented in detail in Fig. 2. SKA, isolated from *hemolytic streptococci* bacteria, can form a complex with the circulating plasminogen called SKA–plasminogen activator complex. SKA promotes thrombolytic, but it does not have specificity to fibrin and in comparison, to t-PA, it may cause antigenic reactions in subsequent uses [46]. Urokinase, termed as urinary-type plasminogen activator (uPA), can reduce the levels of both plasminogen and fibrin and is mainly administered for PE treatment. With considerable fibrinogenolysis which leads to excessive production of FDP, urokinase is far less clinically applicable than other thrombolytic drugs [47]. A new generation of thrombolytic agents have been designed with the aim of improving the specificity and affinity for fibrin and increased half-life. The t-PA class includes alteplase (rt-PA, the recombinant form of the t-PA), tenecteplase (the multi-combination mutant variant of alteplase), reteplase [48].

The main purpose of thrombolytic drugs is to accelerate fibrinolysis by mimicking the endogenous t-PA. In the fibrinolytic cascade, these pharmacological agents can increase the levels of cleaved plasmin, as a proteolytic enzyme, by activating the fibrin-bound plasminogen to the clot fragmentation in large vascular beds, particularly in coronary, cerebral, and pulmonary arteries [49]. Moreover, plasmin has the potential to lyse other circulating proteins such as fibrinogen with lower affinity [50].

A low level of fibrinolysis is observed in patients with ARDS due to the decreased plasmin and increased PAI-1 in plasma and edema fluid of the patients [51, 52]. Preclinical studies have shown that administration of PA decreases coagulation and thrombosis in animal models but at the same time they can promote the inflammatory responses contributing to ALI [53, 54]. Stringer et al. demonstrated thrombolytic medications enhanced the infiltration of neutrophils and, therefore, exacerbated the inflammation which may cause further tissue injury [54]. In another study, the administration of uPA to a mouse model of endotoxemia-induced lung injury enhanced the activation of neutrophils induced by LPS [55, 56]. In a case report of a young patient with rapidly evolving ARDS and refractory to the common treatment, a combination of nebulized rt-PA [21] and its bolus IV dose [17] was administered and followed by 15,000 IU unfractionated heparin per day. They concluded that the combination of nebulized and IV thrombolytic medication has a role for the better management of ARDS [57]. Previously, plasminogen activators have been tried in 20 patients with sepsis or trauma induced ARDS, refractory to the conventional management. They concluded that plasminogen activators are associated with a significant improvement in PaO_2_ with normal coagulation tests and no bleeding [58].

The study by Rehberg et al. further showed that the combination therapy of intravenous recombinant human antithrombin (rhAT), and nebulized t-PA significantly restored pulmonary gas exchange, improved the PaO_2_/FiO_2_ ratio, attenuated pulmonary obstruction and reduced the ventilatory pressures [59].

Hardaway et al*.* have reported that the use of either SKA or urokinase in 18 patients with ARDS secondary to trauma and/or sepsis resulted in an improvement of the partial pressure of oxygen (PaO_2_) [58, 60]. In addition, no bleeding was observed, and clotting parameters persisted normal. These studies suggested the potential of thrombolytic medications in the treatment of coagulation and thrombosis of ARDS patients. However, there is insufficient data about the safety of such therapy, since thrombolytic medications may induce systemic bleeding. Moreover, the results of a systematic review and meta-analysis of 22 preclinical studies on the beneficial effects of thrombolytic agents in animal models suggested fibrinolytic therapy, including t-PA, uPA and plasmin, for ALI by improving lung function and attenuation of inflammatory responses and significant reduction in mortality [61].

**Fig. 3:**
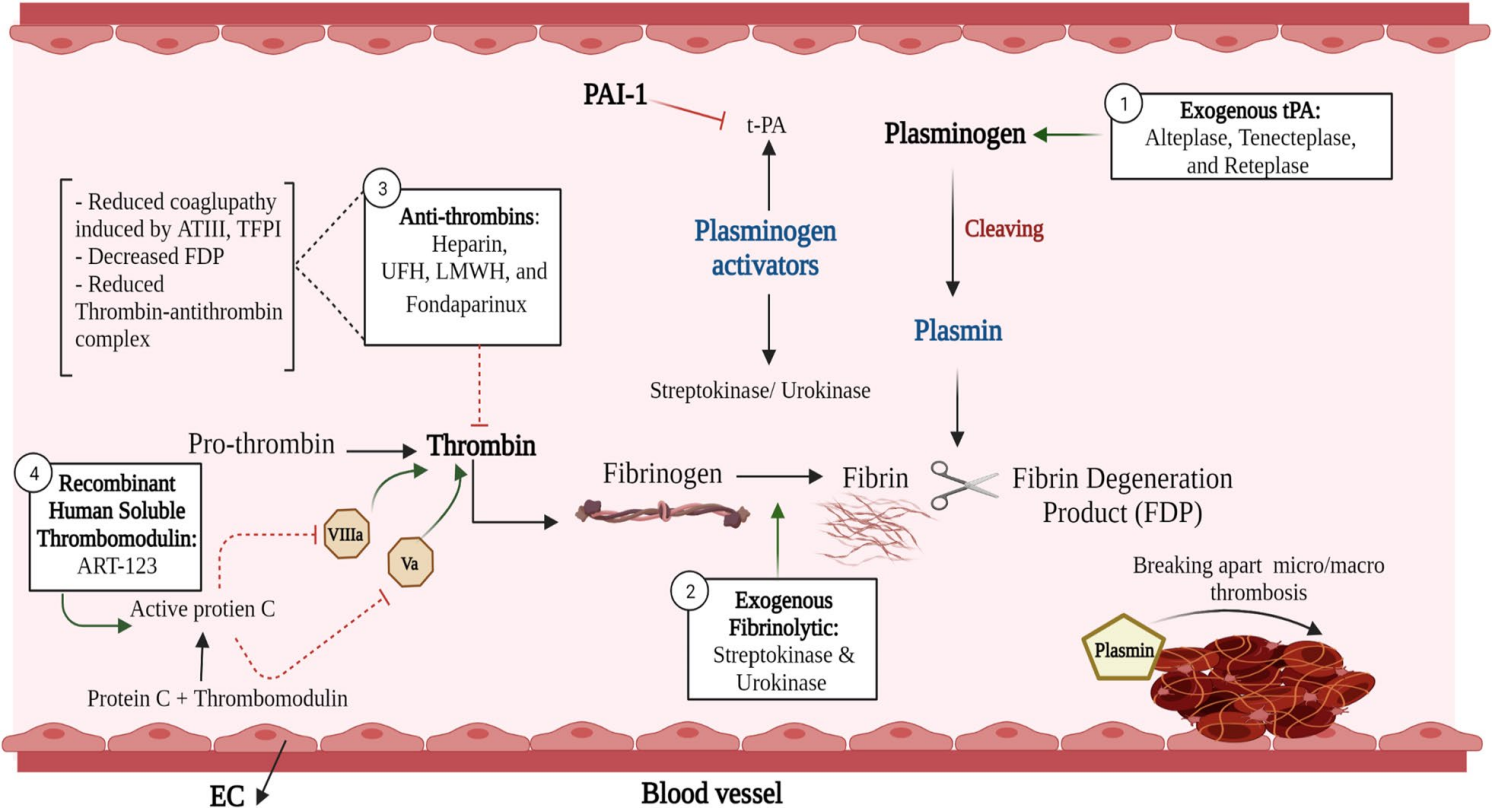
Intravascular thrombin/fibrin formation pathway and related anti-thrombolytic therapy. *Created with BioRender.com

The intravascular thrombin/fibrin formation pathway and related anti-fibrinolytic therapy is presented in Fig. 3.

*Activated protein C (APC)* APC inactivates factors Va and VIIIa through proteolysis and, therefore, suppresses thrombin formation [62, 63]. In several animal models of ALI, the use of APC has been found to reduce pulmonary cell injury, coagulopathy, and inflammation while increasing fibrinolysis [64–67]. Kotanidou et al. found that APC inhalation attenuated the endotoxin-induced lung injury and inflammation The authors suggested that APC acted through modifications of leukocyte trafficking via suppression of VCAM-1 expression. Similar results have been achieved in the study of Slofstra et al. [67] after treating mice with inhaled APC. In this case, APC inhalation significantly reduced the LPS-induced pulmonary inflammation by suppressing neutrophil recruitment, coagulation and inflammatory markers in bronchoalveolar lavage fluid leading to improved lung function. Moreover, Maniatis et al. reported a reduction in inflammation, prevention of endothelial barrier disruption and attenuation of hypoxemia in ventilator-induced lung injury in mice [65]. However, Cornet et al. reported a slight reduction in coagulopathy. No inflammation and bacterial clearance in *Pseudomonas aeruginosa*-induced pneumonia was observed in rats after APC inhalation [66]. Animal models of ALI showed that inhaled recombinant human activated protein C (rh-APC) improves pulmonary oxygenation and attenuates inflammation, with no significant increase risk of systemic bleeding [64, 68].

Despite its promising results in preclinical studies, a phase III trial showed no beneficial effect for APC in patients with severe sepsis [69]. In this randomized multicenter trial on 1697 patients with septic shock, the mortality of patients at 28 and 90 days was not significantly different between the APC and placebo groups.

*Tissue factor pathway inhibitor (TFPI)* TFPI has been used in several preclinical studies for the treatment of lung injury. In 1993, Creasey et al. reported that administration of recombinant TFPI (rTFPI) led to a marked decrease in IL-6 level, attenuated the coagulation response and reduced mortality in the baboon model of septic shock [70]. Specifically, TFPI reduced fibrin thrombi deposition and edema development in the lungs. Histological analysis reported significant protective effects of TFPI in organs such as the liver, kidneys, spleen, adrenals, and gall bladder and a mild effect in the lungs when compared to non-treated animals. Enkhbaatar et al. also showed that pre- or post-treatment with rTFPI reduced pulmonary vascular injury by inhibiting leukocyte activation [71]. rTFPI decreased the levels of TNF-α (as well as gene expression), cytokine-induced neutrophil chemoattractant and myeloperoxidase in lung tissues. In an in vitro assay, rTFPI inhibited TNF-α production in LPS-stimulated monocytes and also significantly inhibited formyl-Met–Leu–Phe-induced neutrophil activation. However, the effectiveness of TFPI in clinical studies of sepsis and severe pneumonia has not yet been proven [72, 73]. In a randomized, multicenter, phase III clinical trial performed on 1784 patients with severe sepsis and elevated international normalized ratio (INR), IV infusion of rTFPI did not show beneficial effects on mortality of patients [72]. In addition, rTFPI infusion was implicated with increased risk of bleeding and a reduction in prothrombin fragment 1.2 and the levels of thrombin antithrombin complex. Another multicenter clinical trial performed in 188 centers monitoring 2138 patients with severe community-acquired pneumonia revealed that treatment with rTFPI did not reduce mortality of the patients [73].

*Thrombin*–*thrombomodulin (TM)* TM, in a recombinant soluble form (ART-123), has been used to improve coagulopathy in preclinical and clinical studies. In the first approach, a bolus of ART-123 was administered to a rat model [74]. Treatment with ART-123 significantly reduced the mortality and liver dysfunction induced by LPS and also inhibited the release of pro-inflammatory cytokines. Furthermore, ART-123 reduced the levels of high-mobility group box-1 protein (HMGB1), which is suggested to be closely related with the development of sepsis. In another study, a bolus IV injection of ART-123 was administered to four healthy male volunteers [75]. The treatment with ART-123 diminished the activity of prothrombinase immediately, 24, and 48 h after administration without affecting thromboelastographic values. A decrease in prothrombin and thrombin–antithrombin complexes has been evidenced in a phase II clinical trial following IV administration of ART-123 to 371 patients with severe sepsis and suspected disseminated intravascular coagulation [76]. However, no differences were observed with respect to the placebo group in inflammatory markers, bleeding, and thrombotic events or in the appearance of new infection. In a multicenter phase III clinical trial, ART-123 was administered daily during 6 days to patients with DIC related to infection or hematologic malignancy [77]. Treatment with ART-123 significantly improved DIC, reduced bleeding and the incidence of bleeding-related adverse events. Previous studies have shown that TM significantly increase the resolution rate of DIC, while the results of two large randomized controlled trials (KyberSept and SCARLET) in septic patients revealed that TM cannot significantly reduce the rate of mortality [78]. In a systematic review, Yamakawa et al. reported that recombinant human soluble thrombomodulin (rhTM) reduced 28–30-day mortality in sepsis-induced DIC patients, while the results of another systematic review showed 13% reduction in mortality that was not significant [79]. Although some favorable effects were suggested, no declarative judgments about rhTM beneficial effects in sepsis-induced coagulopathy can be made from the data available so far.

## Pharmacotherapy strategies affecting coagulation in patient with COVID-19

### Anticoagulant therapy in patient with COVID-19

As coagulopathy is the main pathophysiology characteristic of COVID-19, the use of anticoagulative and thrombolytic agents is considered additive therapeutic options in patients with severe COVID-19. However, although prophylactic doses seem to be recommended in most patients to reduce coagulopathy and microthrombus formation, it is necessary to obtain results from clinical trials to determine the optimal dose that can improve the outcome of the COVID-19 patients [80].

In this regard, heparin for its anticoagulative, anti-inflammatory, and endothelium protective properties has been considered for patients with COVID-19 [81, 82]. In an open-label clinical trial, 1074 patients were randomized to receive either empiric full dose anticoagulation (*n* = 529) or the usual care pharmacological thromboprophylaxis (*n* = 529). The empiric full dose of anticoagulation was considered the dose for the treatment of acute DVT. The patients were evaluated for up to 14 days or recovery that is, discharge from the hospital or no requirement for oxygen supplementation during the last 24 h. The authors concluded that in comparison to the usual care pharmacological thromboprophylaxis, therapeutic doses of heparin do not improve hospital survival or duration of organ support in severe cases of COVID-19 [83]. In an open-label clinical trial, 219 non-critically ill hospitalized patients with COVID-19 were randomized to either receive therapeutic anticoagulation or usual care pharmacological thromboprophylaxis. Their results showed that, in comparison to the usual thromboprophylaxis, therapeutic doses of heparin improved the survival to hospital discharge and duration of organ support in non-critically ill cases of COVID-19 [84]. In a randomized clinical trial on 600 patients with COVID-19 admitted to ICU, the intermediate dose of enoxaparin (1 mg/kg daily) was compared to the standard prophylactic anticoagulation (enoxaparin, 40 mg daily) and the patients were followed up for 30 days. The results of this RCT indicated that intermediate doses of enoxaparin compared with its standard-dose did not have a significant impact on thrombosis, 30-day mortality and the need for extracorporeal membrane oxygenation in critically ill patients with COVID-19. Moreover, intermediate dose of enoxaparin was associated with a significant higher rate of severe thrombocytopenia [85]. Notably, the usual care pharmacological thromboprophylaxis dose of unfractionated heparin is 5000 IU twice daily and enoxaparin 40 mg daily subcutaneously. While the therapeutic doses of heparin is considered an IV bolus of 80 IU/kg, followed by 18 IU/kg/h in a continuous infusion. The therapeutic dosing of enoxaparin is 1 mg/kg twice daily [86].

In a recently performed RCT, Sulodexide, a relatively stronger anticoagulant than enoxaparin, or placebo were administered within 3 days of clinical onset of COVID-19 in outpatients setting. The patients received Sulodexide at the dose of 500 LRU (lipase releasing units) twice daily or placebo for 21 days. The results of this RCT showed that fewer people in the Sulodexide group required hospitalization (*p* = 0.03). The patients in Sulodexide group also had better clinical outcomes, significantly lower D-dimer levels and CRP levels, and needed less supplemental oxygen. However, Sulodexide was not associated with a significant difference in thromboembolic events, major bleeding, or mortality [87]. In a clinical trial on 228 moderately ill hospitalized patients with COVID-19 and elevated levels of D-dimer, the therapeutic doses of heparin was compared to the prophylactic dose regimen. The results of this study showed no difference between two arms of the study in terms of the requirement for admission to intensive care wards or mechanical ventilation. However, therapeutic heparin significantly reduced the odds of all-cause mortality by 78% at 28 days in moderately ill patients, but not in severely critically ill patients [88].

In a randomized, open-label clinical trial on 465 hospitalized patients with moderate severity of COVID-19 and elevated D-dimer levels, the therapeutic dose of heparin was compared to the prophylactic dose. The patients were evaluated for 28 days. The authors of this study reported that the therapeutic dose of heparin was associated with a significant lower odds of death at 28 days. However, therapeutic heparin was not associated with a remarkable change in the primary outcome of the study which was a composite of mortality, rate of mechanical ventilation or admission to ICU [89].

In another randomized RCT on 253 hospitalized adult patients with COVID-19 and at the risk of thromboembolic events, the thromboprophylaxis efficacy of therapeutic dose of heparin was compared to the prophylactic or intermediate dose. The patients were evaluated for 30 days. The results of this RCT demonstrated that therapeutic doses of LMWH was more effective in reducing the major thromboembolism events and mortality rate in hospitalized patients with high levels of D-dimer [90].

A recent online article has reported that heparin can interact with SARS‐CoV‐2 Spike S1 protein receptor binding domain leading to a significant structural change in the protein [91]. In this sense, the use of low-molecular-weight heparin (LMWH) has been recommended for prophylaxis by the International Society on Thrombosis and Hemostasis for all admitted patients with COVID-19 except for cases, where there is a risk of thrombocytopenia. For such patients, fondaparinux has been recommended, since it presents a lower risk of causing thrombocytopenia. The potential benefits of prophylaxis was evidenced in an study reporting a 50% decrease of symptomatic VTE odds in patients receiving a higher dose of thromboprophylaxis heparin [92]. The treatment with a full dose of anticoagulative and thrombolytic agents also could be helpful in COVID-19 experiencing thromboembolic events with an elevated level of D-dimer and high score of sepsis-induced coagulopathy (SIC) [105]. One study showed that the use of heparin in patients with an elevated level of D-dimer (˃ 3.0 µg/ml) and SIC scores ≥ 4 is able to decrease the 28-day mortality [93]. Asakura and Ogawa have recommended the use of heparin and nafamostat, a weak serine protease inhibitor, in the treatment of COVID-19 patients with an elevated level of D-dimer [94]. Taken together, as coagulation plays important role in the progression of DIC associated with severe infection, the use of anticoagulation agents is recommended by many experts and associations [95, 96].

### Thrombolytic therapy in patient with COVID-19

Although the majority of studies have focused on the use of anticoagulation agents in clinical studies, there are few ongoing registered clinical trials for the use of thrombolytic agents for patients with severe COVID-19. Many of these studies are also at the recruitment phase [97–107].

There is preliminary evidence to suggest that thrombolytic therapy such as using t-PA improves the survival rate of critically and mechanically ventilated COVID‐19 patients with ARDS [108]. Wang et al. reported a case series of three patients with COVID-19 induced severe respiratory failure, treated with a loading dose of 25 mg t-PA infusion over 2 h, followed by a 25 mg t-PA intravenously over the subsequent 22 h. All the patients experienced an improvement in their respiratory state with only one of them who survived [108]. In a case series of five critically ill COVID-19 patients with respiratory failure, thrombolytic therapy with alteplase was initiated. All the evaluated patients had initial respiratory and clinical improvement which persisted in three of them. Salvage therapy with alteplase was started at the bolus dose of 50 mg IV infused for 2 h along with heparin drip at rate of 500 U/h during receiving alteplase. In fact, unlike Wang's study, in this case series, higher doses of altplase were administered to patients for a shorter period of time. The patients also received heparin concomitantly. The patients showed a transient recovery of their respiratory status with an improvement in the PaO_2_/FiO_2_ ratio. However, these effects were only durable in one of the patients and lost over time in the other two after completing the t-PA infusion. The authors concluded that additional studies are needed to determine the optimal regimen for t-PA administration with or without anticoagulants or whether additional doses are needed to maintain effects in severe COVID-19 patients with ARDS [109]. In an open-label clinical trial, moderate to severe cases with COVID-19 induced ARDS will be randomized to receive Alteplase 50 mg bolus (10 mg push, 40 mg over 2 h), Alteplase 50 mg bolus plus drip (bolus of 10 mg push, 40 mg over 2 h), then a drip of 2 mg/h over 24 h (total 48 mg infusion) or standard of care. In this study, improvement in PaO_2_/FiO_2_ will be evaluated as primary outcome and National Early Warning Score 2 (NEWS2), National Institute of Allergy and Infectious Diseases (NIAID) ordinal scale, 48 h, 14- and 28-day in-hospital mortality, ICU-free days, coagulation-related event-free days, ventilator-free days, successful weaning from extubation, and survival to discharge as secondary outcomes. This RCT will provide valuable information on the effectiveness, safety and dosing of Alteplase in this population of COVID-19 patients [110]. In a case series of four patients with severe respiratory failure induced by severe COVID-19, t-PA 50 mg was intravenously infused for 2 h. It seems that the respiratory failure, suspected to be caused by pulmonary microthrombi in severe cases of COVID-19, require thrombolysis [111].

It has been illustrated that the use of t-PA in these patients improves oxygenation initially. However, its effects were lost over time after completing the t-PA infusion in some cases. Table 1 summarizes the clinical trials using thrombolytic agents in COVID-19-associated ARDS. Table 2 presents the registered RCTs of thrombolytics for the management of COVID-19. In a pilot open-label RCT, 46 critically ill patients with COVID-19 with elevated D-dimer > 3000 ng/mL and PaO_2_/FiO_2_ ratios < 100 were randomized to receive either recombinant tissue plasminogen activators (rt-PA) (followed by intravenous UFH) or prophylactic anticoagulation with UFH. This pilot RCT could not find any significant difference between groups in terms of PaO_2_/FiO_2_ ratio or SOFA score. However, no major bleeding or thrombotic events were reported in any of included patients [112]. A sub-group analysis was performed on the data obtained from STOP-COVID study and evaluated the effectiveness and safety of t-PA in 59 critically ill patients with P/F ratio < 100. These patients received fibrinolytic therapy with t-PA for pulmonary embolism during 2 weeks of ICU stay. The results of this single arm cohort study did not find any beneficial effects for t-PA in improving oxygenation or hemodynamic parameters [113].

**Table 1:** Summary of clinical studies prescribing thrombolytic agents in COVID-19-associated ARDS

Thrombolytic agents	No. of patients	Study design	Dosage/duration	Main outcomes	References
t-PA	3	Case series	25 mg intravenously (first 2 h) and 25 mg (subsequent 22 h)	Transient improvement in their respiratory status	[108]
Low dose t-PA	3	Case series	30–50 mg	Significant increase oxygenation, off oxygen within 3–7 days	[119]
Plasminogen	13	Clinical trial	10 mg/ twice daily	Increased oxygenation, relief of chest tightness	[117]
t-PA	10,000	Simulation study (Markov model)	–	Reduced mortality (47.6% [t-PA] versus 71.0% [no t-PA])	[120]
Recombinant t-PA, LMWH Enoxaparin, and tocilizumab (anti-IL-6 receptor)	1	A case report	25 mg intravenously (first 2 h) and 25 mg (subsequent 22 h)	resolution of the skin ischemia and cytokine release syndrome (CRS), improved respiratory parameters, no adverse effects	[118]
t-PA	5	Case series	25 mg (first 2 h) + 25 mg (subsequent 22 h) and 50 mg (first 5 h) + 50 mg (subsequent 24 h)	All 5 patients to had an improved respiratory status following t-PA administration	[109]
t-PA	46	Clinical trial	25 mg over 2 h then 25 mg for the next 22 h of drug infusion, immediately followed by UFH	no significant difference between groups in terms of PaO_2_/FiO_2_ ratio or SOFA score, as well as no risk of major bleeding or thrombotic events	[112]
t-PA	59	Cohort	50 mg initial bolus, with the median cumulative dose of 50 mg for the median infusion time of 2 h	No beneficial effects in improving oxygenation or hemodynamic parameters	[113]
t-PA	1	Case report	25 mg (first 2 h) and 25 mg (subsequent 22 h)	The patient’s hemodynamics improved, as well as his hypercapnia, alveolar dead space, and ventilatory ratio	[121]

*t-PA* recombinant tissue-Plasminogen Activator

**Table 2:** Ongoing registered clinical trials of thrombolytics for the management of COVID-19

Thrombolytic	ID	Study type	Recruiting status	Numbers of patients	Population age (years)	Intervention group(s)	Primary outcomes	Secondary outcomes	References
Alteplase	NCT04926428	RCT	Completed	15	18–80	Alteplase	Changes in lung perfusion	Coagulation (changes in D-Dimer, standard coagulation test and fibrinogen), Oxygenation (changes in PaO_2_/FiO_2_)	[98]
Nebulized Alteolase	NCT04356833	RCT	Recruiting	66	16–70	Group 1: 10 mg rt-PA nebulized QID for 14 days, Group 2: 20 mg rt-PA in nebulized TDS for 14 days, Group 3:control	treatment efficacy, Change in PaO_2_/FiO_2_, Safety, fibrinogen levels	lung compliance, Clinical status, SOFA score, oxygen free days, ventilator-free days, ICU stay, incidence and duration of New oxygen via ventilation use, incidence and duration of MV, hospital mortality	[99]
Alteplase	NCT04357730	RCT	Not recruiting	50	18–75	Group 1: Alteplase 50 mg bolus (10 mg push, 40 mg over 2 h) Group 2: Alteplase 50 mg bolus plus drip (bolus of 10 mg push, 40 mg over 2 h), then a drip of 2 mg/h over 24 h (total 48 mg infusion) Group 3: control	PaO_2_/FiO_2_ improvement	PaO_2_/FiO_2_ ≥ 200 or 50% increase in PaO_2_/FiO_2_, NEWS2, NIAID ordinal scale, 48 h, 14 and 28 days in-hospital mortality, ICU-free days, coagulation-related event-free days, Ventilator-free days, Successful and weaning from paralysis extubation, Survival to discharge	[101]
Alteplase	NCT04640194	RCT	Recruiting	270	≥ 18 years	Group 1: low dose of Alteplase plus SOC, Group 2: high dose of Alteplase plus SOC, Group 3: SOC	Time to clinical improvement or hospital discharge	All-cause mortality, ventilator-free days, Improvement of SOFA score, Number of major bleeding events, PaO_2_/FiO_2_ ratio	[108]
Tenecteplase	NCT04505592	RCT	Recruiting	60	18–75	Group 1: tenecteplase 0.25 mg/kg (maximum 25 mg), Group 2: tenecteplase 0.50 mg/kg (maximum 40 mg), Group 3: Control	Number of participants free of respiratory failure, Number of occurrences of bleeding	In-hospital deaths at 14 and 28 days, ventilator-free days, respiratory failure-free days, vasopressor-free days, Vasopressor doses at 24 and 72 h, PaO_2_/FiO_2_ ratio at 24 and 72 h, ICU-free days, Hospital length of stay, new-onset renal failure, need for renal replacement therapy	[102]
Tenecteplase	NCT04558125	RCT	Recruiting	45	18–75	Group 1: Tenecteplase infusion plus SOC, Group 2: Placebo infusion plus SOC	Percent improvement in shock index	Clinical status based upon 7-point scale	[103]
rNAPc2	NCT04655586	RCT	Recruiting	160	18–90	Group 1: high dose of rNAPc2 (loading dose of 7.5 μg/kg SC on day 1 followed by 5 μg/kg SC on days 3 and 5), Group 2: low dose of rNAPc2 (loading dose of 5 μg/kg SC on day 1 followed by 3 μg/kg SC on days 3 and 5), Group 3: Heparin	Change in D-dimer level from Baseline to day 8, or day of discharge, Number of major or non-major clinically relevant bleeding events, Time to recovery within 30 days of randomization	Major or non-major clinically relevant bleeding events, bleeding events, Time to first occurrence of a composite of thrombotic events, all-cause mortality, change in tissue factor, interleukin-6 and high sensitivity C-reactive protein laboratory values	[97]
Defibrotide	NCT04348383	RCT	Recruiting	150	≥ 18 years	Group 1: Defibrotide 25 mg/kg 24 h continuous infusion + SOC, Group 2: Placebo + SOC for 15 days	Clinical improvement on WHO scale	Mortality rate, serious adverse events, clinical improvement by NEWS2 scales, decrease of IL-6 levels, biologic response (lymphocytes count, D-dimer, CRP, LDH, CPK, Ferritin), radiological response	[104]
Defibrotide	NCT04335201	Clinical trial	Recruiting	50	≥ 18 years	Defibrotide 25 mg/kg/day, infusion for 2 h, every 6 h (Defibrotide 6.25 mg/kg each dose) for 7 days	Attenuation of the progression of acute respiratory failure	Adverse events, duration of hospitalization, systemic inflammation, overall survival	[105]
Defibrotide	NCT04530604	Clinical trial	Active, not recruiting	12	18–70	Defibrotide 25 mg/kg/day every 6 h, each dose IV infused over 2 h for 7 days	Number of major hemorrhagic complications during 2 weeks	Overall survival, ventilator free survival, Number of ventilator-free days, improvement in oxygenation, change in the WHO ordinal scale	[106]
Defibrotide	NCT04652115	Clinical trial	Recruiting	42	18–100	Deibrotide IV infusion	The rate of adverse event of special interest (bleeding and hypotension)	–	[107]

*rt-PA* recombinant tissue-Plasminogen Activator, *paO*_*2*_*/FiO*_*2*_ arterial oxygen partial to fractional inspired oxygen, *SOFA* sequential organ failure assessment, *ICU* intensive care, *MV* mechanical ventilation, *ADR* adverse drug reaction, *NEWS2* National Early Warning Score 2, *NIAID* National Institute of Allergy and Infectious Diseases, *SaO*_*2*_ oxygen saturation, *SOC* Standard of Care, *rNAPc2* recombinant nematode anticoagulant protein c2, *CRP* C-reactive protein, *LDH* lactate dehydrogenase, *CPK* creatine Phosphokinase

In a severe COVID-19 patient with highly developing ARDS, sepsis and massive pulmonary embolism, alteplase was administered at the dose 100 mg slow IV infusion as rescue therapy. It seems that thrombolytic medications is a potential critical care practice for life-threatening pulmonary embolism in COVID-19 Pneumonia [114].

One must also be very careful with this strategy because of the risk of catastrophic bleeding associated with t-PA. In another case series report, t-PA (30–50 mg) was administered to three patients with severe ARDS with continuous worsening of respiratory failure despite being on therapeutic anticoagulation [115]. The patients presented with a significant increase in oxygenation and dyspnea, which was evident earlier when compared to other patients admitted with COVID-19 in the ICU with similar severe condition but without receiving t-PA infusion. Moreover, the patients were on room air after 3–7 days of t-PA infusion and discharged within 2 weeks after negative COVID-19 PCR. The authors proposed that the administration of 50 mg t-PA over 3 h is adequate for patients who meet: FiO_2_ requirement ≥ 0.7, PaO_2_/FiO_2_ ratio < 100, D-dimer > 1000 ng/ml, no contraindications of thrombolysis and no additional causes for worsening their condition such as secondary infection or fluid overload. The inhalation of freeze-dried plasminogen was investigated in 13 patients with COVID-19 ARDS [116]. Plasminogen at the dose of 10 mg was administered twice daily for severe/critical COVID-19 subjects (*n* = 8), and once daily for moderate COVID-19 subjects (*n* = 5). The treatment improved lung lesions determined with CT scan, increased oxygen saturation and slowed down heart rates. The authors concluded that plasminogen treatment may be used at a larger scale, particularly to COVID-19 patients with clinical conditions quickly changing from moderate to severe conditions. A different approach using decision analytic Markov state transition model was developed to simulate the evolution of critically ill COVID-19 patients [117]. In this model, the simulation of t-PA administration using a total of 10,000 patients led to reduction in mortality (47.6%), suggesting that t-PA may improve the recovery of critically ill patients and reduce COVID-19-associated mortality. Finally, another case report described the combined administration of thrombolytic (25 mg of rt-PA during 2 h, followed by additional 25 mg over the subsequent 22 h, and LMWH) and immunosuppressive (400 mg of tocilizumab, an anti-human IL-6 receptor monoclonal antibody) therapy to a critically ill COVID-19 patient [118]. After the treatment, the patient experienced a significant improvement in respiratory parameters and in skin ischemia and a resolution of cytokine release syndrome without evidence of adverse effects. The authors concluded that the combined administration of rt-t-PA/tocilizumab could be taken into consideration, since it led to transient respiratory stabilization and resolution of the cytokine storm and skin manifestations. Furthermore, Omarjee proposed the use of ticagrelor to treat patients with COVID-19 as it stops platelet function and aggregation, neutrophil extracellular traps (NETs) release, and vascular permeability [93].

### Thrombolytic therapy: pharmacotherapy considerations

Since the local (in the lung) and systemic regulation of fibrinolysis is impaired in COVID-19, targeting this pathway using thrombolytic agents can be considered in COVID-19-associated ARDS. However, most of the clinical information regarding the administration regimen was extrapolated from other approved indications and animal studies. The first challenge lies in selecting appropriate thrombolytic agent. Although their mechanisms of action are the same, there are some differences in fibrin specificity and complications. None of the thrombolytic agents showed complete lysis of the fibrin clot and fibrin specificity. The specificity of SKA for fibrin is low, while that of Alteplase and Reteplase is moderate, and Tenecteplase shows most fibrin specificity [122–124]. Along with class effect difference, safety profile, and appropriate administration time are unanswered challenges.

#### Patient and time selection

There is insufficient evidence to determine which patients with ARDS and at which time would benefit from thrombolytics. Based on prior studies, thrombolytics has been administrated in severe ARDS patients with PaO_2_/FiO_2_ ≤ 100. However, the time of administration is not well defined. Although it is hypothesized that earlier administration of thrombolytics before developing severe cases of the ARDS and diffuse development of endothelial damage can prevent its extension of ARDS, the clinical data is scarce.

Base on pathogenesis category, three different phases including exudation, proliferation, and fibrosis were overlapped in ARDS. Impaired fibrinolysis by upregulating tissue factor, downregulating activated protein C, blockage of fibrinolysis due to levels of PAI-1 and thrombin-activatable fibrinolysis inhibitor excess was prominent in the pathogenesis of ARDS during the exudative phase. Consequently, pharmacotherapy by thrombolytics in this phase lead to suppression of thrombosis in situ propagation. On the other hand, it showed that at the beginning of endothelium injury, the development of microthrombi and fibrin deposition intend to prevent the systemic invasion and limit expansion of leakage in damaged endothelium and epithelium [125].

In hence, a key concept to determine which fibrin formation is unregulated or works as a protective barrier would be a guide for inclusion of patients for thrombolytics therapy. Most of clinical study of thrombolytic includes severe hypoxemia intubated patient with PaO_2_/FiO_2_ ≤ 100. However, in a previous study, abnormal alveolar hemostatic balance was observed in non-intubated patients with pneumonia as well as patients with ARDS [126].

However, early thrombolytic therapy in patient with localized lung thrombosis or microvascular thrombosis may be considered to prevent uncontrolled coagulation cascade and cross talking of inflammation and coagulation. Elevated serum levels of C-reactive protein (CRP), ferritin and D-dimer can predict mortality and poor outcome, but is not helpful in selecting the appropriate patient and timing of thrombolytic administration due to unavailable cutoff points and its prognostic nature versus therapeutic markers. Along with considering the severity of PaO_2_/FiO_2_, elevated CRP levels (as it is cheap and available), recognition of therapeutic endothelial biomarkers and evaluation of pulmonary dead space can help select the right patient in a right timing. Obviously, the time period in which thrombolytic medications are beneficial is limited and they should be prescribed at the appropriate time to be effective and with the least risk of bleeding and complications. Although biomarkers such as CRP might be applicable, surrogates that can indicate the endothelial injury may allow more specific pharmacotherapy of ARDS patients.

#### Rout and dose of administration

Another important dilemma is the dosage regimen with an acceptable safety profile. As shown in Table 1, most studies used a low dose of Alteplase which is close to the dosing of thrombolytic therapy in pulmonary embolism, so little is known on the efficacy and/or safety of higher doses of alteplase. Regarding the improvement of oxygenation, the duration of improvement was short lived. Thus, it is not clear whether repeated doses or continuous infusion may lead to long-lasting effects. Unlike the pathophysiology of pulmonary embolism, over-activation of clot formation, in addition to suppression of fibrinolysis, leads to the propagation of pulmonary endothelial injury and the vicious cycle proceeds. In this way, the continuous effect of fibrinolysis needs to prevent the deposition of fibrin in the alveolar space and make breakdown of hypercoagulable state in situ and systematically. In an animal model of ARDS, nebulized t-PA improved the oxygenation; however, the beneficial effect of nebulization or intrapulmonary administration is not clear. The combination of the systemic route and pulmonary delivery of thrombolytic agents provides an option to overcome the short duration of gas exchange improvement. However, a clinical study evaluating this combination therapy is lacking. Whereas, pulmonary system has many potentials in drug delivery, the lung delivery strategies have the advantage of reduction in systemic toxicity and enhancing drug efficacy. Intravenous administration of Recombinant tissue plasminogen activators (rt-PA) and its nebulization was previously studied [57]. In patients with plastic bronchitis, instillation and nebulization of Alteplase resulted in fibrin degradation and successful treatment of patients [127, 128].

Although, local pulmonary drug therapy such as nebulization is an alternative and safe rout of drug delivery to the lung, as shown in Table 1, most studies used systemic delivery of Alteplase. Contrary to some concern regarding minimizing the use of nebulization in era of COVID-19 pandemic, because of elevated risk of infection spread, nebulization can be considered an alternative rout of thrombolytics to reduce systemic side effects along with maintaining more duration of response. Based on previous studies, the gas exchange improvement was seen in short lasting after termination of systemic alteplase [108, 115].

Insomuch, repeated administration or high dose of systemic alteplase intensifies the risk of serious complications, nebulization of thrombolytics could be considered a means of drug delivery. Although, special pulmonary formulation of the alteplase has been developed (BioTherapeutics, LLC, Denver, CO, and HTD Biosystems, Inc., Hercules, CA), animal experiments showed the efficacy and stability of alteplase in form of nebulization [45].

In conclusion, further studies should evaluate the efficacy of alteplase in different routes of administration to justify the optimum dose and means of administration. Nevertheless, based on the limited studies in non-ARDS patients, we recommend a dose of 5–12 mg alteplase at interval of 2–6 h as a possible nebulization regimen for this thrombolytic drug.

### Complication of the thrombolytic therapy

Another important issue regarding thrombolytics in ARDS related COVID-19 is the safety considerations. The most worrying issue in prescribing thrombolytic therapy is the undesirable bleeding complications, especially because of the narrow therapeutic window of these compounds. The use of thrombolytics for VTE has been associated with an increased risk of major and intracranial bleedings. Although the rate of fatal and major bleeding in reperfusion therapy for pulmonary embolism was reported less than 5% and 10–20%, respectively, the frequency of bleedings may vary for different population of ARDS patients with COVID-19, and heterogenicity of ARDS.

It has been previously shown that the rate of intracerebral hemorrhage (ICH) in patients who received systemic thrombolytics for acute pulmonary embolism was higher in comparison with the rate of ICH for acute coronary reperfusion, depending on the patient risk factors and the route of administration [128, 129].

Considering different populations in COVID-19, diffuse organ involvement, inflammatory cytokine state, administration of other medications along with the patient risk factors may elevate the risk of bleeding during thrombolytic therapy. Because of the limited sample size of studies in this field, the estimation of incidence of bleeding complications and comparison with patients who received therapy due to pulmonary embolism is not supported [130].

Beyond the thrombolytics, the imbalance between fibrinolysis and thrombin production, hyperfibrinogenemia and thrombocytopenia in COVID-19 may accompanied with local thrombosis in lung along with bleeding tendency in other organs simultaneously [131].

Identified risk features including history of intracranial hemorrhage, neoplasm, ischemic stroke or head trauma, structural cerebrovascular lesions, suspected aortic dissection, active peptic ulcer or active bleeding except for menses, uncontrolled hypertension, major surgery (< 3 weeks), concomitant anticoagulants use, and pregnancy should be considered before administration of thrombolytics [123, 124, 132]. Other risk factor for bleeding such as age > 65 years, cancer, renal and liver failure, thrombocytopenia, diabetes, anemia, antiplatelet therapy, reduced functional performance, low body weight, female gender, use of nonsteroidal anti-inflammatory drugs (NSAIDs), antiplatelet and anticoagulants potentiate the probability of bleedings. Due to paucity of clear benefit of thrombolytics in era of ARDS associated with COVID-19, it is reasonable to consider thrombolysis for selected younger patients at low risk of bleeding. Regardless of the route of thrombolytic administration, routine lab data for monitoring of CBC diff, INR, PT, and PTT should be considered. Improving the patient consciousness and tight monitoring for sign of bleeding should be carried out to prevent unwanted adverse effects.

#### Pharmacodynamics and pharmacokinetics

Although alteplase has been studied more than any other thrombolytics in ARDS, the published evidence of pharmacodynamic, catalytic activity and pharmacokinetics of thrombolytics in ARDS is scarce. Since resistance to thrombolytics such as alteplase was reported in prothrombotic and pro-inflammatory state, likely due to cytokine release syndrome, COVID-19 might be associated with resistance to thrombolytics and consequently, less efficacy might be observed [133].

Previously it was showed that increased activity of PAI-1, high levels of factor XIII, dense clot structure and rebound hypercoagulability state increased the probability of resistance to clot dissolution by thrombolytics. Moreover, the effect of acute inflammatory state, endotheliitis, hepatic, renal, and cardiac dysfunction, and elder age may have an influence on the pharmacodynamic effects of alteplase which has not been studied in the population of patients with COVID-19-associated ARDS.

Based on previous pharmacokinetic studies of alteplase in acute myocardial infarction (AMI) and stroke, the half-life of alteplase is less than 5 min, the volume of distribution is approximately similar to plasma volume which could be explained by a 2-compartment model and it is metabolized by the liver [134].

Furthermore, the pathophysiological differences between AMI, stroke and COVID-19-associated ARDS may cause different responses to t-PA regimen, so making it difficult to directly apply the PK/PD findings of previously mentioned population in patients with ARDS. The use of t-PA in COVID-19-associated ARDS results in transient improvement in oxygenation [108]. Ongoing fibrin formation and different PKPD data may also have an impact on short duration of improvement. In other words, thrombolytic drugs have been used for a short period of time in previous studies and after the initial improvement in oxygenation, this effect did not last [108]. The explanation for this could be that this hypercoagulability state constantly produced thrombotic factors.

Taken together, further PK/PD studies of t-PA in COVID-19-associated ARDS with focused on clinical outcomes could determine the appropriate dosing and means of thrombolytics administration in era of ARDS.

## Conclusions

Considering that despite timely appropriate modalities, the COVID-19 pandemic is still spreading with high morbidity and mortality rate, COVID-19 requires novel therapeutic approaches to minimize its complications. It is presumed that COVID-19 comprised of three critical clinicobiological steps: initial, propagating, and complicating phases. Given the lungs of patients with ARDS are in a prothrombotic state, elevated hepatic production of fibrinogen in plasma and lung epithelium as an acute-phase protein, damage to the lung surfactant, and fibrin deposits in the intra-alveolar spaces, anti-thrombolytics seem to have a potential role in the complicating phase 3 of the disease by dissolution of microthrombosis and the extra-vascular and intra-alveolar fibrin deposition in alveolar space. Inhaled or intravenous administration of these agents seems to be a salvage therapy for severe ARDS associated with COVID-19 by prompt attenuation of lung injury. Considering the pathogenesis of COVID-19-related ARDS and mechanism of action of thrombolytic agents, thrombolytics appear attractive options in stable patients without contraindications. Tenecteplase, the most highly fibrin specific with low risk of bleeding, high percentage of success, and single-dose administration has been the mainstay of thrombolytic therapy and can be offered to patients with COVID-19-related ARDS.

### Future prospects in COVID-19-associated coagulopathy

Further studies are demanded to determine whether the observations in these studies are reliable or not. Well-designed clinical trials with large sample size will determine whether thrombolytics lead to improvement in PaO_2_/FiO_2_ ratio, weaning from the ventilator, and the extent of bleeding risk from these medications. The optimal treatment regimens and doses, either alone or in combination with other therapeutic anticoagulants should also be investigated in future studies.

## Data Availability

Not applicable.

## Abbreviations

ARDS: Acute respiratory distress syndrome
ALI: Acute lung injury
DIC: Intravascular coagulation
PIC: Intravascular coagulopathy
CRP: C-reactive protein
ACE2: Angiotensin-converting enzyme 2
TMPRSS2: Transmembrane protease serine 2
CXCL10: C–X–C motif chemokine ligand 10
MCP-1: Monocyte chemotactic protein-1
MIP-1α: Macrophage inflammatory proteins-1α
NF-κB: Nuclear factor kappa B
STAT3: Activator of transcription 3
TLR: Toll-like receptor
NK: Natural killer
VTE: Venous thromboembolic
aPTT: Activated partial thromboplastin time
PT: Prothrombin time
DAMPs: Damage-associated molecular patterns
MCP-1: Monocyte chemoattractant protein-1
TF: Tissue factor
FDP: Fibrin degradation product
VWF: Von Willebrand factor
t-PA: Tissue-type plasminogen activator
PAI-1: Plasminogen activator inhibitor-1
DVT: Deep vein thrombosis
PE: Pulmonary embolism
PA: Plasminogen activator
uPA: Urokinase-type
t-PA: Tissue-type
uPA: Urinary-type plasminogen activator
SKA: Streptokinase
rhAT: Recombinant human antithrombin
PaO_2_: Partial pressure of oxygen
APC: Activated protein C
VCAM-1: Vascular cell adhesion molecule 1
ERK: Extracellular-signal-regulated kinase
TFPI: Tissue factor pathway inhibitor
LPS: Lipopolysaccharide
INR: International normalized ratio
TM: Thrombin–thrombomodulin
HMGB1: High-mobility group box-1 protein
LMWH: Low-molecular-weight heparin
SIC: Sepsis-induced coagulopathy
NEWS2: National Early Warning Score 2
NIAID: National Institute of Allergy and Infectious Diseases
NETs: Neutrophil extracellular trap
CRP: C-reactive protein
ICH: Intracerebral hemorrhage
NSAIDs: Nonsteroidal anti-inflammatory drugs
AMI: Acute myocardial infarction
